# Hypothesis-free analyses from a large psoriatic arthritis cohort support merger to consolidated peripheral arthritis definition without subtyping

**DOI:** 10.1007/s10067-017-3637-2

**Published:** 2017-04-22

**Authors:** Daniel Stekhoven, Almut Scherer, Michael J. Nissen, Véronique Grobéty, Nikhil Yawalkar, Peter M. Villiger, Burkhard Möller, on behalf of the Swiss Clinical Quality Management for Rheumatic Diseases

**Affiliations:** 1 SCQM Statistics Group, Zurich, Switzerland; 2 NEXUS Personalized Health Technologies, ETH Zurich, Zurich, Switzerland; 3 Department of Rheumatology, University Hospital Geneva, Geneva, Switzerland; 4 Department of Rheumatology, Immunology and Allergology, Inselspital, University Hospital Bern, Freiburgstraße, 3010 Bern, Switzerland; 5 Department of Dermatology, Inselspital, University Hospital Bern, Bern, Switzerland

**Keywords:** Anti-TNF, Disease activity, Peripheral arthritis, Psoriatic arthritis, Subtypes

## Abstract

Current ClASsification criteria for Psoriatic ARthritis classification criteria for psoriatic arthritis (PsA) provide a preliminary definition of inflammatory articular disease. This study aimed to further characterize PsA peripheral arthritis using purely data-driven approaches for the affected joint distribution pattern. PsA patients from the Swiss Clinical Quality Management in Rheumatic Diseases (SCQM) database were clustered according to similarities in 66 swollen and in 68 tender joints. Clusters were compared in terms of other disease characteristics and studied for coincidence with traditional PsA subtypes, stability over time and treatment response upon first tumour necrosis factor alpha (TNF-α) therapy. Clustering of 957 patients resulted in an oligoarticular, a polyarticular hand dominated, a polyarticular foot dominated and a fourth cluster which was characterized by polyarticular involvement of the hands and feet. Of the traditional PsA subtypes, only a non-PsA-specific oligoarticular joint involvement pattern was retrieved by clustering. When comparing clusters in other disease manifestations, only minor and clinically probably irrelevant differences occurred. Over time, clusters were more robust than traditional PsA subtypes. Patients in different joint clusters had similar response rates upon first anti-TNF-α therapy, and minimal disease activity was achieved in 56% of 285 patients, irrespective of cluster membership. Hypothesis-free approaches to group PsA patients yield clusters with improved consistency, but without clinically important differences. Taken together, the current peripheral arthritis definition by GRAPPA without further specification into subtypes is strongly supported by the data.

**Electronic supplementary material:**

The online version of this article (doi:10.1007/s10067-017-3637-2) contains supplementary material, which is available to authorized users.

## Introduction

Psoriatic arthritis (PsA) is typically an autoantibody negative, chronic inflammatory disease of synovial joints, peritendinous tissues and entheses, usually in association with psoriatic skin or nail disease. Both psoriatic skin and joint disease have a strong genetic background [1], which appear to be different in some important gene loci of immune response regulation [2]. Furthermore, another recent study indicated some genetic differences in relation to the PsA phenotype [3], but the complexity of PsA manifestations is not fully explained by current genetic data. Tumour necrosis factor alpha (TNF-α) and different cytokines of the IL-23/IL-17 axis currently are established treatment targets in PsA [4].

Moll and Wright were the first to describe a set of more or less typical traditional PsA subtypes [5], which were later defined in more detail for research purposes [6]. Distal interphalangeal (DIP) peripheral joint involvement alone, often in conjunction with psoriatic nail disease [7], is considered to be specific enough to allow for PsA diagnosis [8]. In contrast, other PsA subtypes share dominant features with other rheumatic entities, e.g. axial disease with other types of spondyloarthritis (SpA), or symmetric polyarticular disease with rheumatoid arthritis (RA). Symmetry in joint involvement is a function of its number [9]. The more joints involved are in PsA, the more resemble PsA disease manifestations RA. Furthermore, at an individual time-point, a single patient may satisfy several different subtypes and a patient may switch from one subtype to another over time [10].

Following a currently discussed anatomy-based hypothesis, unknown site-specific factors may link DIP manifestations to inflamed anchorage tendon fibres between the psoriatic nails, the finger extensor tendons and the periosteum [11–13]. In contrast, large joints, metacarpophalangeal (MCP) joints or proximal interphalangeal (PIP) joints, which are typically involved in oligoarticular or symmetric polyarticular PsA, would lack a direct connection to the nails, thereby suggesting that other pathologies may be dominant in these subtypes. In another current disease concept, arthritis may, in analogy to the Koebner phenomenon of psoriatic skin, be triggered by repeated microtrauma in genetically or otherwise susceptible individuals, thereby activating locally accumulated immune cells in an autoinflammatory fashion [14]. Assuming highly individual movement behaviours, a huge heterogeneity in joint involvement patterns would be the consequence of this disease model.

While the debate about the existence and relevance of PsA subtypes for the clinics is ongoing [10, 15], details of peripheral joint involvement were not included in the current broad preliminary ClASsification criteria for Psoriatic ARthritis (CASPAR) definition of inflammatory articular disease, but postponed to the research agenda [16]. In the present study, we utilized a fully data-driven approach in order to obtain any characteristic peripheral joint involvement pattern. We aimed specifically to reproduce the traditional subtypes as far as defined on a peripheral joint basis. In their absence, we would consider equivalence of peripheral arthritis in any location for disease definition. Consequently, the current preliminary definition of peripheral joint PsA without specification in its localization would gain additional support [5, 16].

## Methods

### Patients and setting

This study was nested in the Swiss Clinical Quality Management in Rheumatic Diseases (SCQM) program in rheumatic diseases. All 957 patients with a definite diagnosis of PsA according to the CASPAR classification criteria were included into analysis [16]. This study was performed in compliance with the Helsinki Declaration from 1964. Patients provided written informed consent prior to inclusion. Ethical approval for patient enrolment into the SCQM program and related studies was obtained from the Swiss Academy of Medical Sciences review board. Patient registration in SCQM is restricted to board-certified rheumatologists. Data for this study were collected from 1997 until May 2015. More than 65% of the rheumatologists, who contributed data on PsA patients, were from private practices.

### Disease variables

Peripheral joint manifestation data consisted of ‘involved’/‘not involved’ for both swelling and tenderness in each joint of the 66/68 joint status [17]. Descriptive data and the data for the cluster analyses were obtained at the same time-point unless otherwise stated. Traditional PsA joint involvement groups were defined according to a later specification of the Moll and Wright criteria as ‘oligoarthritis type’ in the case of less than five affected joints, the ‘DIP joint predominance type’ in the case of >50% of the total joint count being DIP joints, and a ‘symmetric type’ was defined when >50% of joints in non-oligoarticular and non-DIP type patients were involved in a symmetrical manner [6].

Dichotomous stand-alone variables, human leukocyte antigen (HLA)-B27 status, CASPAR classification items (current psoriasis, personal or family history of psoriasis, history of dactylitis, enthesitis, axial disease, nail manifestations and rheumatoid factor status) were collected from any visits since inclusion. Disease-defining radiographic data were assimilated from the local evaluations of the treating rheumatologists. Time-dependent dichotomous data included current treatment with non-steroidal anti-rheumatic drugs (NSAIDs), corticosteroids, synthetic long-acting anti-rheumatic drugs (DMARD) and anti-TNF-α agents. Current psoriatic skin activity was repeatedly assessed on a 7-point Likert scale, and had to be documented on at least one occasion as being mild or more severe, in order to satisfy the current skin psoriasis criterion in the CASPAR classification criteria [16]. Patient and physician global assessment of disease activity and patient pain were collected on 10-point Likert scales. Time-dependent continuous parameters included erythrocyte sedimentation rate (ESR), C-reactive protein (CRP), the disease activity score in 28 joints (DAS-28) [18], the Stanford health assessment questionnaire of disability index (HAQ-DI) [19], the Short-Form Health Survey (SF-36) and the dermatology life quality index (DLQI) [20].

### Minimal disease activity

We compared the clusters for the proportion of patients achieving a status of minimal disease activity (MDA) at 12 months (±3 months) after start of the first documented anti-TNF-α therapy [21]. If multiple visits were available in the time-window, the visit closest to 12 months was chosen. If no visit was available within this time window, the last visit between 2 and 9 months after the start of ongoing anti-TNF-α treatment was utilized. Absent or almost absent current psoriasis skin activity on the 7-point-scale substituted for the missing Psoriasis Area and Severity Index (PASI) [22]. In order to compensate for missing repeated assessment of enthesitis scores, which were according to OMERACT 8 only defined as not mandatory domains [23], we approximated ‘possibly in MDA’ on the basis of four fulfilled out of the six remaining domains. ‘Definitive MDA’ was defined using fulfilment of five out of the six repeatedly completed domains. In a sensitivity analysis, we calculated MDA in five out of seven domains for patients with absent enthesitis at any completed visit. Furthermore, an additional sensitivity analysis, using an enlarged response population by including 126 subjects with probable PsA according to the medical diagnosis of the treating rheumatologist, was performed.

### Statistics

Swollen joint data was chosen as the primary variable to be analysed in a data-driven strategy of hierarchical clustering given the more objective nature of this variable compared with the tender joint data. The Manhattan distance, simply counting the number of joints that are differently involved or not involved in two patients, was used as metric and complete linkage clustering method, due to its property of deriving compact clusters. We compared this method with other established clustering approaches, but found none of them to be more suitable. In secondary analyses, patients were analysed with the same statistical procedures based on their tender joint data.

Patient transitions between joint involvement clusters or traditional PsA subtypes were predicted using k-nearest neighbour classification (k = 10, no constraints) trained on the data as assigned to the respective clusters at inclusion and predicted 1 year later.

The effect of the cluster membership on MDA after start of a first anti-TNF-α therapy was assessed using logistic regression. We assessed the changes in single disease activity outcome measures using an analysis of covariance with robust regression for non-normally distributed scale-based single disease activity outcome measures in a completers-only approach. Improved involvement on a single joint level was evaluated using Fisher’s exact test. Multiple testing correction was performed either using the methods of Bonferroni or Benjamini-Hochberg [24] when indicated. A 5% significance level was defined. All analyses were performed in the statistical software ‘R’.

## Results

### Patient clustering on peripheral swollen joints

**Fig. 1:**
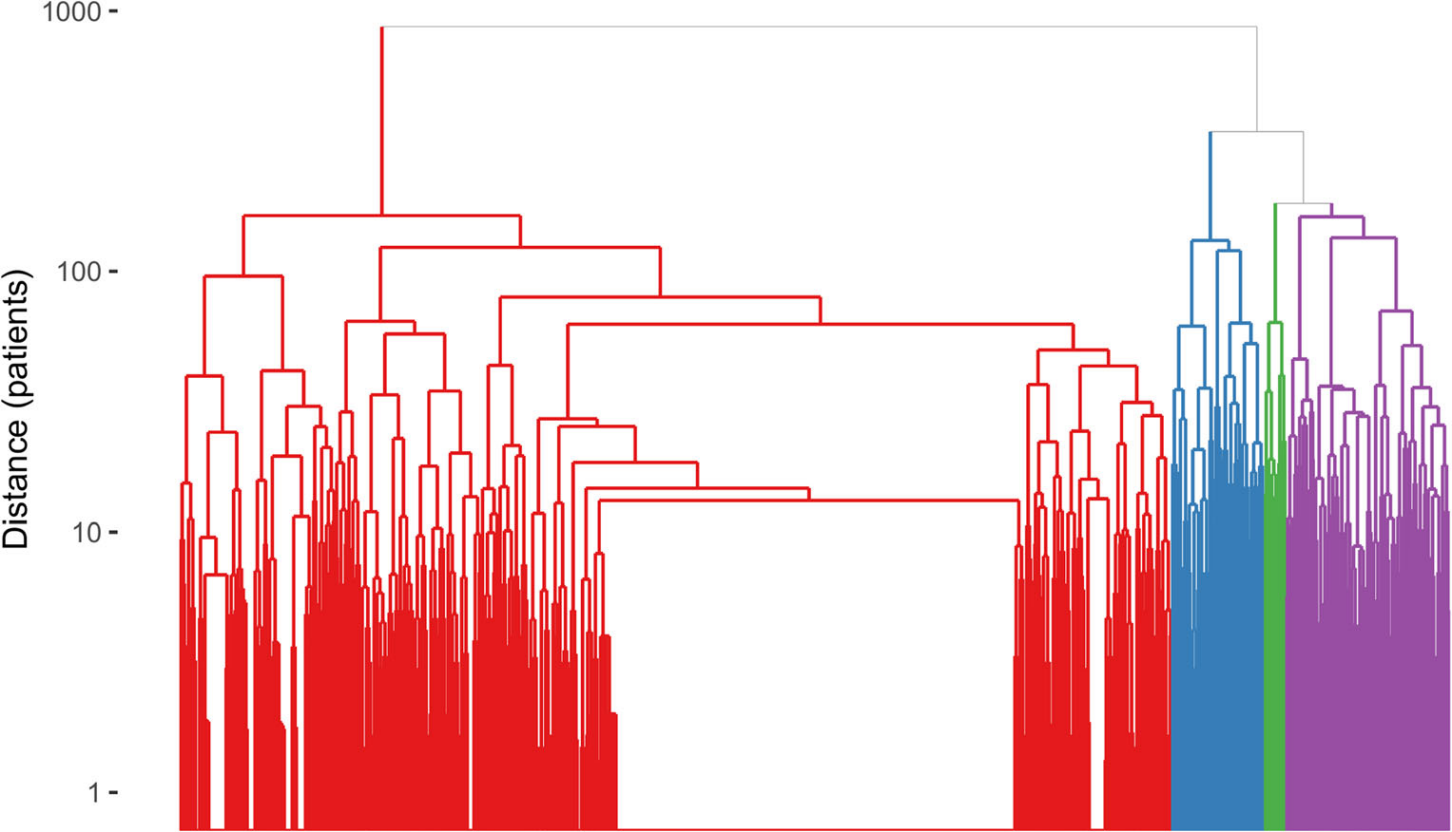
Unpruned dendrogram of hierarchical clustering on the swollen joint manifestations in patients with definite PsA [16]. The first split occurs between the large oligoarticular cluster (*red*) and the rest. Then, at a distance of about 350, the feet-dominated cluster (*blue*) is separated from the rest, followed by the split into the polyarticular (*green*) and hand-dominated clusters (*purple*) at a distance of about 170 (colour figure online)

The hierarchical clustering was optimized to yield a feasible number of groups, which resulted in four clusters. A detailed description of the clinical status at inclusion of all 957 patients with definite PsA [16] grouped into these four clusters is provided in Table 1. The dendrogram illustrates that the largest cluster of 748 patients separated first from the other patients (Fig. 1). Swollen joints in this cluster could be at any location, but with a median of one swollen joint (IQR 0–4), the numbers were characteristically low. This ‘oligoarticular’ cluster also included patients with currently absent or inactive peripheral arthritis. Next, a cluster of patients with predominant feet involvement separated (*n* = 70). The final split occurred between the patients with hand-dominant arthritis (*n* = 123) and a so-called polyarticular cluster composed of patients with many swollen joints at their hands and feet (*n* = 16). By cluster definition on tender joint data, 71% of all subjects were assigned to the same cluster as defined by swollen joints. In a sensitivity analysis, repeating the same statistical operations on swollen and tender joint data in the anti-TNF-α-naïve patients at inclusion (*n* = 464), clusters with the same four qualities were retrieved (data not shown).

**Table 1:** Descriptive statistics of clusters based on swollen joint data for patients with CASPAR-positive definite PsA at inclusion

	Oligo *N* = 748	Poly *N* = 16	Hands *N* = 123	Feet *N* = 70	*P* value
Female	46%	31%	46%	39%	0.46
Age	48 (40–57)	55 (50–58)	50 (44–60)	45 (41–54)	0.004
Disease duration	6 (2–13)	4 (2–8)	6 (2–11)	5 (3–8)	1
Arthritis ever	92%	100%	100%	100%	0.0005
Enthesitis ever	63%	70%	67%	60%	0.79
Spinal disease ever	43%	38%	41%	42%	0.95
Current skin psoriasis	83%	94%	91%	97%	0.001
Psoriasis patient history	97%	100%	98%	97%	0.85
Psoriasis family history	35%	13%	38%	51%	0.05
Nail psoriasis ever	29%	17%	32%	33%	0.87
Negative RF ever	97%	90%	99%	100%	0.28
Dactylitis ever	66%	94%	81%	91%	0.0005
Radiographic criterion	31%	33%	39%	33%	0.63
Oligoarticular	70%	0%	2%	*6%*	0.0005
DIP-predominant	3%	0%	3%	0%	0.55
Any DIP involvement	11%	38%	31%	40%	0.0005
Symmetric polyarticular	15%	100%	57%	57%	0.0005
Inflammatory back pain ever	34%	50%	33%	31%	1.00
HLA-B27-positive	10%	6%	7%	9%	0.45
Anti-TNF-α	52%	38%	54%	50%	0.09
DMARD	63%	81%	65%	71%	0.25
Corticosteroids	9%	19%	20%	13%	0.0085
NSAID	52%	88%	63%	80%	0.0005
SJC66	1 (0–4)	24 (17–34)	10 (4–12)	13 (2–17)	<0.0001
SJC28	1 (0–2)	20 (10–22)	7 (0–10)	4 (0–7)	<0.0001
CRP [mg/l]	6 (2–9)	11 (8–29)	8 (4–17)	8 (5–16)	0.0018
ESR [mm/h]	10 (5–20)	29 (15–59)	14 (6–25)	16 (11–36)	0.0001
Physician global	3 (2–5)	7 (6–8)	5 (3–7)	5 (4–7)	<0.0001
Patient global	4 (2–7)	3 (3–7)	6 (3–7)	7 (4–8)	<0.0001
Patient pain	5 (2–7)	3 (3–9)	6 (3–8)	6 (4–8)	<0.0001

Data are from the visit used for cluster analysis unless otherwise stated. *P* values are from Fisher’s exact test for nominal variables or a Wilcoxon rank-sum test for continuous/ordinal variables. No multiple testing correction was performed. For continuous variables, medians and interquartile ranges (in parentheses) and, for nominal variables, proportions (in %) are reported

*CRP* C-reactive protein, *DIP* distal interphalangeal, *DMARD* synthetic disease-modifying anti-rheumatic drugs, *ESR* erythrocyte sedimentation rate, *NSAID* non-steroidal anti-inflammatory drugs, *RF* rheumatoid factor, *SJC* swollen joint count on the basis of 28/66 joints

### Comparison of clusters for other disease characteristics

When comparing obtained clusters for other clinical disease features, patients were significantly older in the polyarticular and hand-dominated clusters. Skin psoriasis was currently less active, and current DIP predominant joint involvement as well as dactylitis in the patients’ past history were significantly less frequent in the oligoarticular cluster compared with the other clusters. In contrast, no differences were detected among the clusters for disease-defining psoriatic nail pathologies, spinal involvement, enthesitis or the presence of characteristic radiological features of PsA. The full set of comparisons between the four swollen joint clusters is reported in Table 1.

As 29% of patients were allocated to another cluster on the basis of tender joint data compared with swollen joint data, tender joint clusters and their comparisons differed in some parameters from those performed on swollen joint clusters, e.g. for enthesitis prevalence. These data are reported in Supplementary Table 1. All the subsequent analyses were performed for both, swollen and tender joint clusters. For simplicity, as both clusters led to comparable results, we will only present the swollen joint cluster data.

### Comparing mathematical clusters with predefined peripheral joint PsA subtypes

**Fig. 2:**
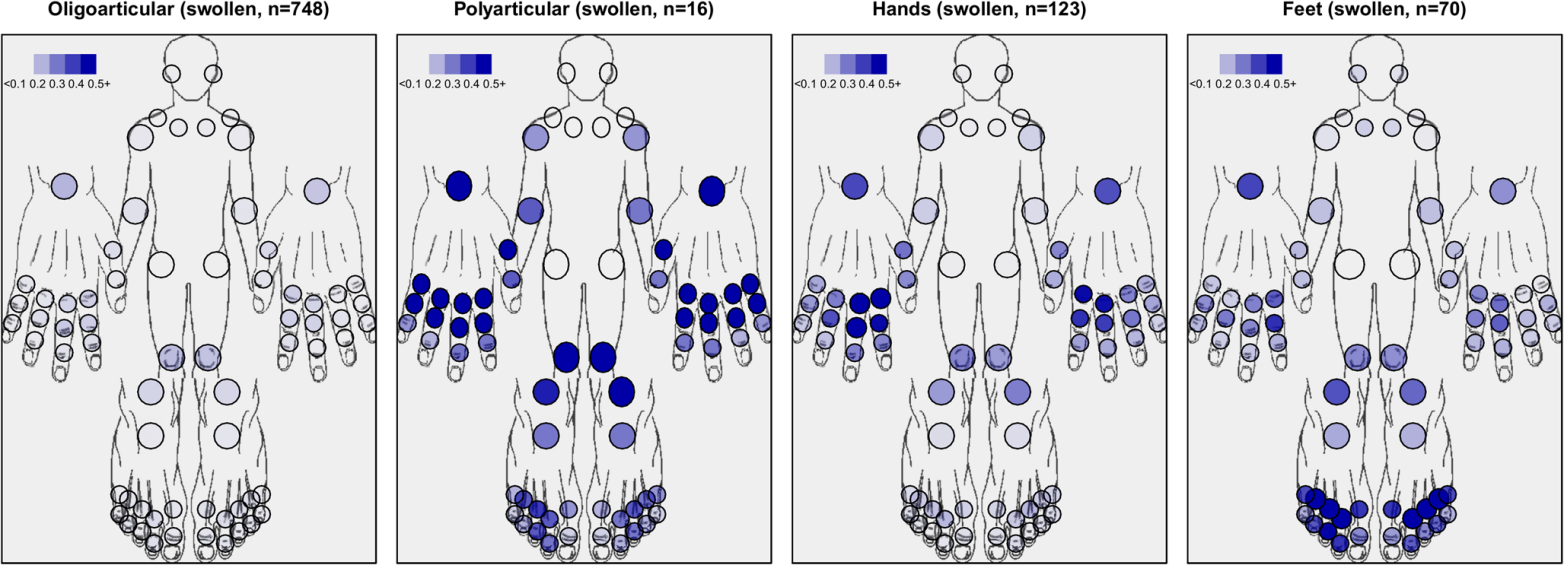
Characterization of the four clusters with respect to joint involvement patterns in the swollen data. *Colour strength* indicates proportion of patients with respective involved joint

Seventy percent of patients in the oligoarticular cluster formally fulfilled the traditional oligoarticular PsA subtype definition [6], but the other, more PsA characteristic traditional subtypes were not retrieved by clustering the swollen joints. DIP joints were less frequently involved than the MCP or PIP joints in any swollen joint cluster (Fig. 2). DIP predominance was only retrieved in secondary tender joint analyses among patients with hand-dominated joint involvement (Supplementary Fig. 1). Also, symmetric tender joint features were overrepresented in the hand-dominated and polyarticular clusters.

### Stability of clusters and traditional PsA subtypes over time

**Fig. 3:**
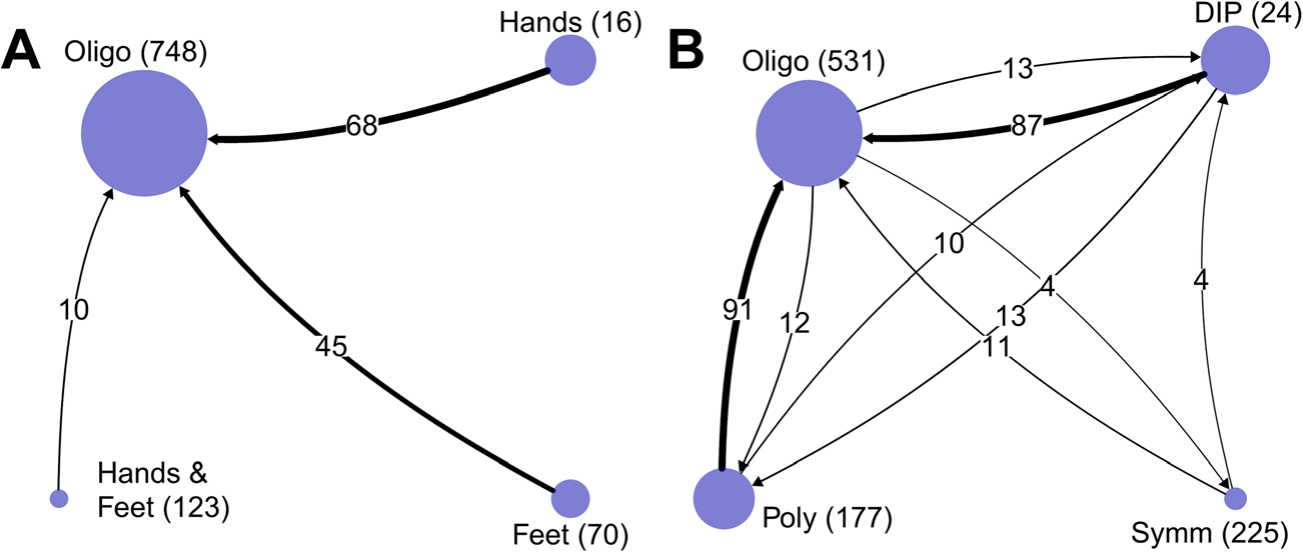
Transition of patients between clusters (**a**) and traditional PsA subtypes (**b**) going from inclusion to follow-up. *Arrows* indicate direction of transition as well as number. *Circles* indicate the size of the respective clusters at inclusion. Absolute numbers at the respective start points are given in parentheses. The ‘Hands & Feet’ circle corresponds to the ‘polyarticular cluster’ from the text

Follow-up data of this cohort was obtained from a median of four visits (IQR 3–6) collected over a median of 3.1 years (IQR 1.5–5.3). We investigated whether patients remained in their initial allocated clusters at the 1-year follow-up visit. There was unidirectional migration towards the oligoarticular cluster in 13% of patients (Fig. 3a). In contrast, bidirectional migration was observed in 26% of cases between the traditional PsA subtypes (Fig. 3b). These observations were confirmed in clusters as well as traditional subtypes when based on tender joint data, and in all subgroup analyses limited to anti-TNF-α-naïve patients (*n* = 464), to only patients with axial involvement (*n* = 397) and to only patients with dactylitis (*n* = 664, data not shown).

### Response to anti-TNF-α therapy in joint clusters

**Fig. 4:**
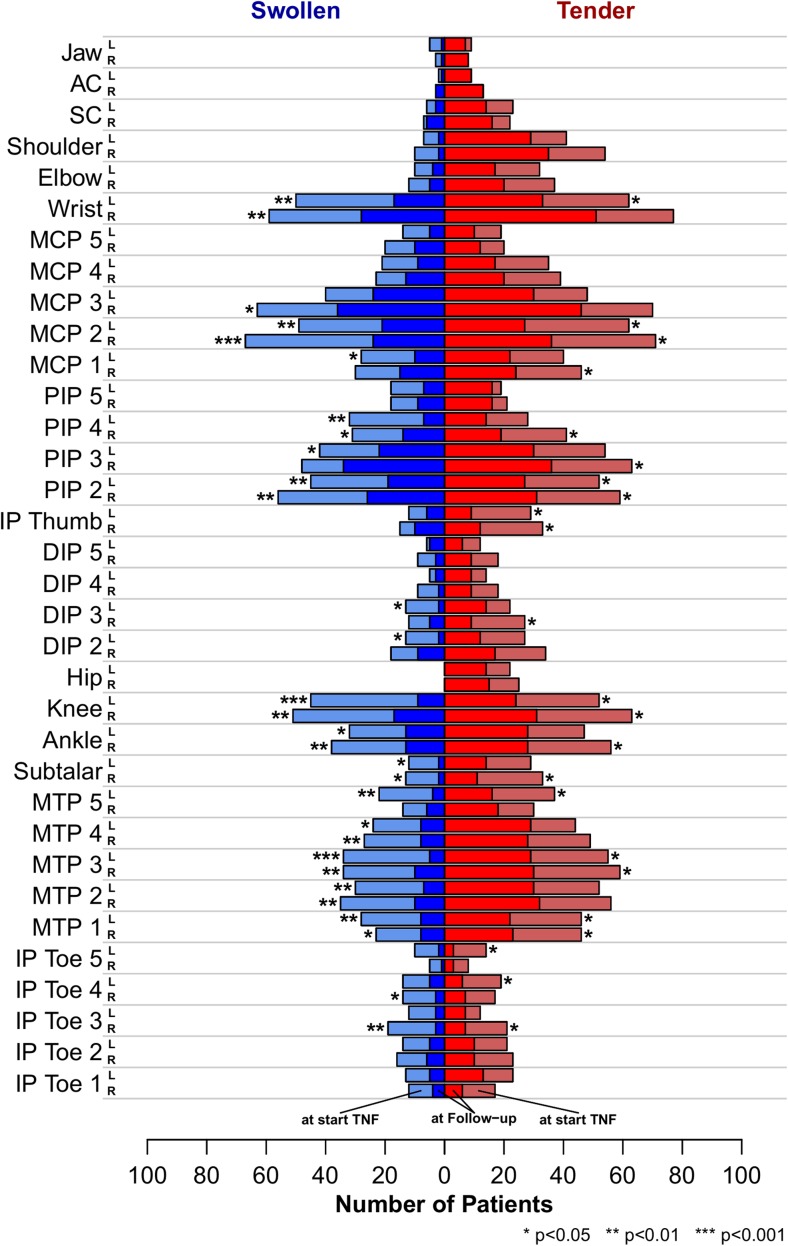
Counts of joint swelling and tenderness before and after anti-TNF-α treatment. Joint swelling (*blue*) or tenderness (*red*) before start of anti-TNF-α treatment (*lighter colours*) and frequency of joint involvement at follow-up visit (*darker*). *Stars* indicate level of significance from a per location Fisher’s exact test. False discovery rate was controlled using Benjamini-Hochberg correction (colour figure online)

Clinical response to treatment was examined in 285 patients with follow-up data at 12 ± 3 months after initiation of their first documented anti-TNF-α therapy. This population was representative of the entire study population with regards to observation time, visit counts and all reported disease parameters. One hundred seventeen (56%) patients were possibly in a state of MDA according to the 4/6 rule, and 78 (38%) patients were definitely in a state of MDA according to the 5/6 rule, at the time of follow-up. The proportion of patients demonstrating possible or definite MDA among the clusters ranged from 35 to 58%. No significant effect of the joint clusters on achieving MDA was found by logistic regression analysis irrespective of the rule of MDA definition. In the sensitivity analysis only including enthesitis-negative patients, 74 patients (41%) achieved MDA with similar proportions in all the clusters (39–50%). Cluster allocations had no significant effect on the evolution of any of the tested single-response items upon first anti-TNF-α therapy in a robust ANCOVA after multiple testing correction (Table 2). The same was true for clusters on tender joint basis (data not shown). As some swollen joint clusters in CASPAR-positive patients were too small for a definitive statement, the population was enlarged to a total of 411 patients, resulting in 20 subjects in the smallest cluster, by including formally CASPAR-negative patients. Negative classification was most frequently caused by missing data. All these individuals had PsA according to the medical diagnosis of the treating rheumatologist. Still, cluster membership on swollen joints remained an insignificant parameter for the results obtained in these secondary anti-TNF-α response analyses (data not shown). Finally, we looked at the responsiveness to TNF blockade for single joints in the original CASPAR-positive cohort. A median of 64% of swollen and 47% of tender joints improved upon anti-TNF-α therapy with only some quantitative difference in dependence of their localization (Fig. 4). Response rates upon anti-TNF-α therapy in DIP joints were similar to other PsA joint manifestations.

**Table 2:** Response to treatment with anti-TNF-α after 1 year in the four clusters

	Oligo *N* = 207	Poly *N* = 8	Hands *N* = 41	Feet *N* = 29
SJC 66	−1 (−3–0)	−10 ([−16]–[−2])	−7 ([−9]–[−1])	−11 ([−17]–[−6])
TJC 68	−1 (−4–0)	−6 ([−8]–[−3])	−5 ([−12]–[−1])	−11 ([−19]–[−5])
Current skin psoriasis	−1 (−2–0)	0 (0–0)	−1 (−2–0)	−1 (−2–0)
Patient global	−2 (−5–0)	−1 (−1–0)	−2 (−3–1)	−4 ([−7]–[−2])
Patient pain	−2 (−4–0)	−1 ([−2]–[−1])	−2 (−3–0)	−2 ([−6]–[−1])
HAQ-DI	0 (−1–0)	0 (0–0)	0 (−1–0)	0 (−1–0)
DLQI	−1 (−6–0)	No data	−1 ([−5]–[−1])	0 (0–0)
SF-36 PCS	5 (0–12)	5 (0–9)	7 (−1–9)	10 (1–13)
SF-36 MCS	1 (−2–5)	−2 (−9–4)	5 (0–9)	8 (0–11)
Physician global	−2 (−3–0)	−3 ([−4]–[−2])	−3 ([−5]–[−1])	−3 ([−6]–[−2])
CRP [mg/l]	0 (−6–0)	−2 (−15–0)	0 (−8–0)	−4 (−12–0)
ESR [mm/h]	−4 (−14–0)	−6 ([−16]–[−2])	−2 (−6–2)	−8 ([−31]–[−2])
DAS28 (CRP)	−1 (−2–0)	−1 (−2–0)	−1 (−2–0)	−2 (−2–0)

Median decrease and interquartile ranges (in parentheses) of different response parameters from start of anti-TNF-α treatment to 1 year later in the four clusters. None of the investigated response criteria showed significant change in a robust ANCOVA after multiple testing correction. *SJC* swollen joint count on the basis of 66 joints, *TJC* tender joint count on the basis of 68 joints, *HAQ-DI* health assessment questionnaire disability index, *DLQI* Dermatology Life Quality Index, *SF-36 PCS/MCS* short-form health survey physical/mental component score

*CRP* C-reactive protein, *ESR* erythrocyte sedimentation rate, *DAS28 (CRP)* disease activity score based on 28 joints and CRP

## Discussion

In this study, we aimed to further detail the current arthritis definition without subtyping given in the CASPAR classification criteria by stringent application of data-driven statistical methods in one of the few currently available large PsA cohorts with a full status including 66/68 joints [8, 16, 25]. This study confirmed the reported large variability of involved joints in their number and location. We found moderate to good agreement between swollen joint and tender joint cluster definitions, but were unable to satisfactorily reproduce the traditional PsA subtypes. We found improved robustness of the clusters over time as compared to previous subtype definitions, but clusters had no other clinically relevant differences than their peripheral joint status. Furthermore, cluster stability over time was still insufficient for a rational basis of true sub-entity definition in PsA.

A precise definition of arthritis in PsA purely with regards to the presence or absence of tenderness and swelling has its limitations [17, 26]. Available alternatives with a higher level of precision would be X-ray studies, which do not cover early disease phases. Evaluation studies on more precise imaging modalities necessary to detect early pathologies and to clearly distinguish arthritis from joint-adjacent enthesitis are still under investigation [27]. Moreover, more sophisticated methods such as high-resolution MRI or ultrasound would hardly be feasible due to the many locations, the variability and the large number of individuals to be studied without prior preparation using rather crude clinical assessments.

Despite the restriction of clinical assessors to board-certified rheumatologists as required by CASPAR [16], with at least 1 year of post-graduate Rheumatology education in a large teaching or university hospital, the setting of many patients coming from outside specialized PsA research centres might raise concerns about the validity of the data. For instance, 70% cumulative dactylitis prevalence over time is higher than expected, which may indicate some uncertainties in the assessment or overestimation of this important disease symptom in routine daily practice. On the other hand, being closer to the ‘real world’ may also be considered as a strength, as long as central data validation by review of each single item of the CASPAR classification criteria compensated for major issues [28]. The association of psoriatic nail and DIP involvement could not be addressed with only general data on nail pathologies [7, 29]. Restriction of response data to the PsA core set domains according to OMERACT 8 [23] in the SCQM registry without repeated enthesitis scores and skin assessment on only ordinal data level caused calculable imprecisions for MDA calculation.

Patients could be included into the SCQM registry at any time after diagnosis. The patients in this study had a tendency towards shorter disease duration and lower median joint counts than in the few other large cohorts with available reports on the 66/68 joint status [10, 16, 25]. TNF-α inhibition was usually started in monotherapy, or as add-on to methotrexate (MTX), which was the DMARD of choice in 69% of cases. Limited patient numbers or lower baseline disease activity prevented other response analyses, but we assume that MTX had, if at all, only a limited effect on the clinical presentation and study outcome [30].

We could exclude significant effects on the peripheral arthritis clusters by anti-TNF-α in treatment-specific subgroup analyses.

There has been some controversy about whether more detailed subtypes in PsA should be maintained or whether division should be restricted to only two subtypes of peripheral and axial disease [10]. Subtype definitions should be as distinct as diagnoses in general; they should be mutually exclusive and, ideally, collectively exhaustive. When applying these requirements to the traditional PsA subtypes, oligoarthritis cannot, strictly speaking, be considered PsA-specific. Furthermore, many cases of polyarticular joint involvement in the present study resembled the 1987 ACR RA criterion of symmetry, while others did not, thereby dissecting another traditional PsA subtype by data analysis. In addition, patients with the most characteristic DIP predominance, or with at least some DIP involvement for definition, were scattered among all the mathematically defined clusters, and the majority of these cases fell into the same mathematically defined hand cluster as the traditional polyarticular symmetric subtype. Taking all these comparative observations in clusters and in traditional subtypes together, with bi-directional migration in the latter, data-driven approaches did clearly not support the traditional peripheral joint-based PsA subtypes.

With the exception of dactylitis frequency, which is by definition not unrelated to peripheral joint involvement, we found no other significant differences between the obtained clusters. More importantly, from a clinical point of view, we observed no significant differences in achieving MDA upon anti-TNF-α therapy, the current first choice of target-specific treatment in PsA [31]. Often, harder joint capsules upon palpation even in actively inflamed DIP joints in PsA may give rise to suggest different pathologies in these than in others such as MCP joints. However, similar improvement rates upon anti-TNF-α therapy in the different localizations may indirectly support appropriate DIP arthritis assessment being truly inflammatory. Furthermore, obtained improvement rates were clearly superior to those observed in osteoarthritis [32, 33].

Thus far, cluster membership of peripheral joints would currently have no clinical implications on the treatment choice. Without a principle reservation of possible differential therapeutic effects on different joints for future treatment modalities, it appears unnecessary to develop subtype-specific treatment response criteria on the basis of peripheral joint involvement. This statement does not exclude the usefulness of subtyping of PsA patients according to more general domains such as axial disease or enthesitis. Authors of a recent abstract came to a similar conclusion [34].

In summary, we found relevant conceptual issues regarding traditional PsA subtypes, but were not able to offer better data-driven alternative-defining subtypes on the basis of peripheral joint involvement. Ongoing research may lead in the future to meaningful subtype systematics on another basis, e.g. by morphology or genetics. Thus far, the present data support the current designation of peripheral arthritis without further specification.

## Electronic supplementary material

Supplementary Figure 1 Characterization of the four clusters with respect to joint involvement patterns in the tender data. Colour strength indicates proportion of patients with respective involved joint (GIF 64 kb)

High resolution image (TIFF 3750 kb)

Supplementary Table 1 (DOCX 19 kb)
