# Supplementary figures and images for: Hypothesis-free analyses from a large psoriatic arthritis cohort support merger to consolidated peripheral arthritis definition without subtyping
Source: Clin Rheumatol. 2017 Apr 22;36(9):2035–43. doi: 10.1007/s10067-017-3637-2 (PMC5554477; doi:10.1007/s10067-017-3637-2)

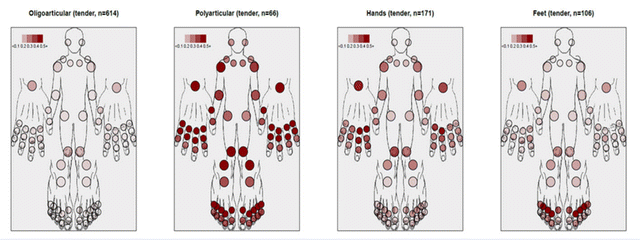

Supplement: Supplementary file 1 — Characterization of the four clusters with respect to joint involvement patterns in the tender data. Colour strength indicates proportion of patients with respective involved joint (GIF 64 kb) [file 10067_2017_3637_Fig5_ESM.gif]

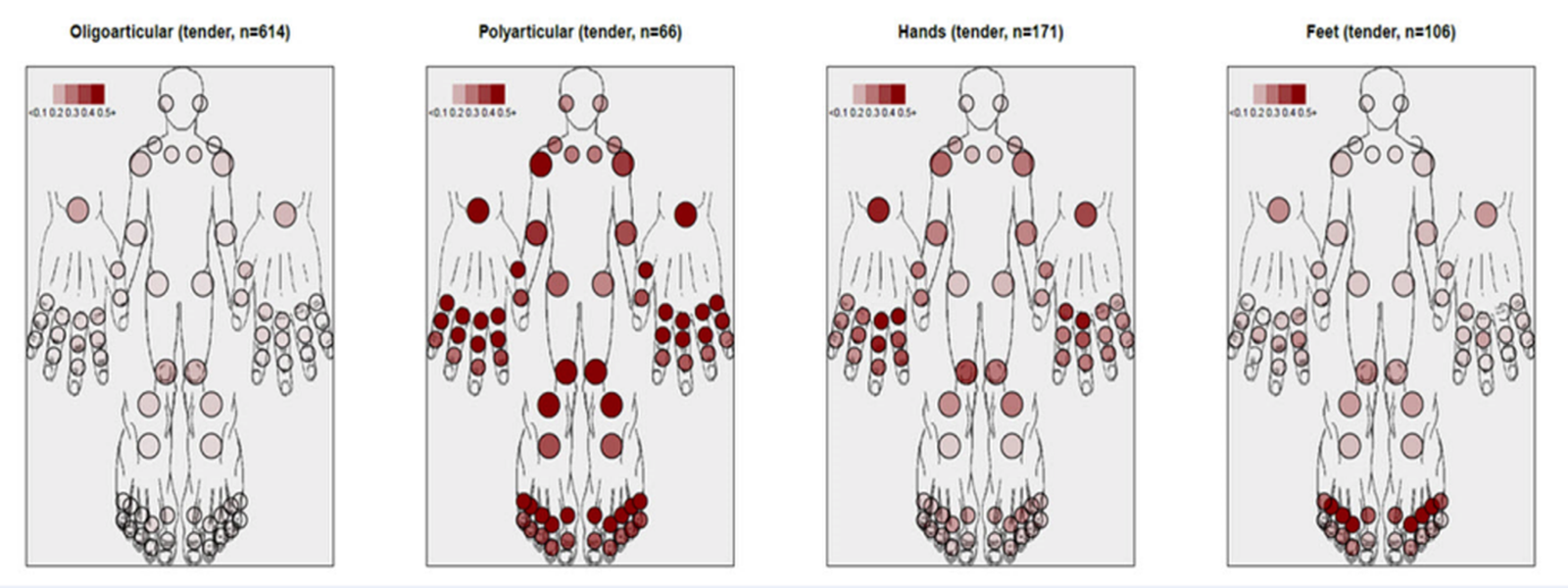

Supplement: Supplementary file 2 — High resolution image (TIFF 3750 kb) [file 10067_2017_3637_MOESM2_ESM.tif]
