# Supplementary material for: Hypothesis-free analyses from a large psoriatic arthritis cohort support merger to consolidated peripheral arthritis definition without subtyping
Source: Clin Rheumatol. 2017 Apr 22;36(9):2035–43. doi: 10.1007/s10067-017-3637-2 (PMC5554477; doi:10.1007/s10067-017-3637-2)
Supplement: Supplementary file 3 — (DOCX 19 kb) [file 10067_2017_3637_MOESM1_ESM.docx]

Supplementary Materials

**Supplementary table 1**

Descriptive statistics of cluster populations based on tender joint data for patients with CASPAR-positive definite PsA at inclusion. P-values are from a Fisher’s exact test for nominal variables or a Wilcoxon rank sum test for continuous/ordinal variables. No multiple testing correction was performed. For continuous variables medians and interquartile ranges (in parentheses) and for nominal variables proportions (in %) are reported.

CRP: C-reactive protein. DIP: distal interphalangeal. DMARD: synthetic disease modifying antirheumatic drugs. ESR: erythrocyte sedimentation rate. NSAID: nonsteroidal anti-inflammatory drugs. RF: rheumatoid factor. TJC: swollen joint count on basis of 28/68 joints.

|  | Oligo  N=614 | Poly  N=66 | Hands  N=171 | Feet  N=106 | p-value |
| --- | --- | --- | --- | --- | --- |
| Female | 43% | 48% | 50% | 46% | p= 0.36 |
| Age | 49 (40-57) | 50 (43-56) | 52 (43-60) | 45 (38-55) | p<0.0001 |
| Disease duration | 6 (2-12) | 7 (2-11) | 6 (2-13) | 5 (3-12) | p= 1 |
| Arthritis | 92% | 98% | 96% | 98% | p= 0.0045 |
| Enthesitis | 60% | 84% | 67% | 64% | p= 0.002 |
| Spinal disease | 40% | 54% | 47% | 47% | p= 0.07 |
| Current skin psoriasis | 83% | 91% | 87% | 89% | p= 0.22 |
| Psoriasis patient history | 97% | 98% | 97% | 97% | p= 0.98 |
| Psoriasis family history | 35% | 32% | 39% | 43% | p= 0.40 |
| Nail psoriasis | 31% | 30% | 24% | 26% | p= 0.61 |
| Negative RF | 97% | 95% | 98% | 97% | p= 0.70 |
| Dactylitis | 65% | 83% | 75% | 86% | p= 0.0005 |
| Radiographic criterion | 29% | 36% | 43% | 34% | p= 0.03 |
| Oligoarticular subtype | 65% | 0% | 1% | 13% | p= 0.0005 |
| DIP predominant | 1% | 0% | 5% | 0% | P= 0.002 |
| Any DIP involvement | 10% | 52% | 45% | 21% | p= 0.0005 |
| Symmetric polyarticular | 19% | 95% | 70% | 47% | p= 0.0005 |
| Inflammatory backpain | 29% | 45% | 41% | 39% | p= 0.22 |
| HLA-B27 positive | 11% | 5% | 8% | 9% | p= 0.70 |
| Anti-TNF-α | 52% | 55% | 48% | 51% | p= 0.38 |
| DMARD | 63% | 64% | 63% | 73% | p= 0.25 |
| Corticosteroids | 10% | 26% | 13% | 8% | p= 0.002 |
| NSAID | 52% | 71% | 58% | 65% | p= 0.004 |
| TJC68 | 2 (0-4) | 29 (24-38) | 12 (8-16) | 9 (6-12) | p<0.0001 |
| TJC28 | 1 (0-2) | 15 (10-22) | 8 (5-11) | 2 (0-4) | p<0.0001 |
| CRP [mg/l] | 6 (2-9) | 8 (4-16) | 8 (3-14) | 8 (4-11) | p= 0.0018 |
| ESR [mm/h] | 10 (5-20) | 18 (6-40) | 12 (6-26) | 13 (8-26) | p= 0.00014 |
| Physician global | 3 (1-4) | 7 (5-8) | 5 (3-7) | 4 (3-6) | p<0.0001 |
| Patient global | 4 (2-6) | 7 (5-8) | 6 (3-8) | 6 (4-8) | p<0.0001 |
| Patient pain | 4 (2-6) | 7 (5-8) | 6 (4-7) | 6 (4-8) | p<0.0001 |
